# Crosstalk between Cu(i) and Zn(ii) homeostasis *via* Atx1 and cognate domains†
†Electronic supplementary information (ESI) available: Materials and methods, a figure showing gel filtration data and a table containing crystallographic data collection and processing statistics. See DOI: 10.1039/c3cc42709aClick here for additional data file.



**DOI:** 10.1039/c3cc42709a

**Published:** 2013-08-07

**Authors:** Adriana Badarau, Arnaud Baslé, Susan J. Firbank, Christopher Dennison

**Affiliations:** a Institute for Cell and Molecular Biosciences , Medical School , Newcastle University , Newcastle upon Tyne , NE2 4HH , UK . Email: christopher.dennison@ncl.ac.uk

## Abstract

The copper metallochaperone Atx1 and the N-terminal metal-binding domain of a copper-transporting ATP-ase can form tight Zn(ii)-mediated hetero-complexes in both cyanobacteria and humans. Copper and zinc homeostasis could be linked by metal binding to these CXXC-containing proteins.

## Abstract 2

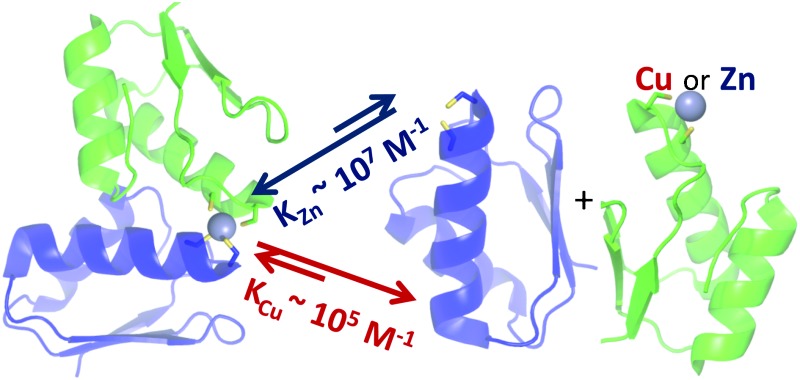
The copper metallochaperone Atx1 and the N-terminal metal-binding domain of a copper-transporting ATP-ase can form tight Zn(ii)-mediated hetero-complexes in both cyanobacteria and humans.

A widely used intracellular copper-trafficking route involves the soluble metallochaperone Atx1 and a P-type ATP-ase. These copper transporters possess one or more N-terminal metal binding domains (MBDs) structurally similar to Atx1 in having a M/IXCXXC metal-binding motif anchored on a ferredoxin (βαββαβ) fold.^1^ For all copper transporters studied, at least one MBD forms a detectable complex with Atx1 in the presence of Cu(i).^2^ Dissociation constants for Cu(i)-mediated hetero-complexes are ∼10 μM or higher,^2b,d^ so at low μM protein concentrations they are only transiently formed. Copper transfer can still occur when complex formation is even weaker.^2c,e,3^ In this study we have found, using both cyanobacterial and human proteins, that the interaction between Atx1 and MBDs of copper transporters is preferentially stabilized by Zn(ii) compared to Cu(i). CXXC-containing copper-trafficking sites are able to bind Zn(ii) at metal and protein concentrations that are thought to be physiologically relevant. This has important implications for assessing potential crosstalk between copper and zinc homeostasis in cells.

The cyanobacterium *Synechocystis* PCC 6803 requires copper in its thylakoids for photosynthesis and respiration, and a copper-trafficking pathway involving Atx1 and the P-type ATPase PacS has been identified.^4^ In the same organism, zinc export occurs *via* the P-type ATPase ZiaA.^5^ PacS and ZiaA each possess a single N-terminal MBD (PacS_N_ and ZiaA_N_, respectively). The Cu(i) affinities of copper and zinc trafficking proteins in *Synechocystis* are all within one order of magnitude at pH 7.0 and copper transfer between Atx1 and the MBDs occurs as expected based on affinities.^3*a*^ The Zn(ii) affinity of ZiaA_N_ is up to two orders of magnitude higher than those of PacS_N_ and Atx1.^3*a*^ The Cu(i) affinities of the trafficking sites in *Synechocystis* are at least six orders of magnitude greater than their Zn(ii) affinities,^3*a*^ but Atx1 has been proposed to be able to bind zinc *in vivo*.^6^ The Cu(i) affinity of the human copper metallochaperone HAH1 is similar to those of the MBDs of the Menkes (MNK) and Wilson (WLN) ATPases,^7^ but Zn(ii) affinities of these proteins have not been reported. MNK^8^ and WLN^9^ can both bind Zn(ii). The *in vivo* (yeast two-hybrid) interaction between HAH1 and the fourth MBD of WLN (WLN4) depends on zinc concentration, and a tight *in vitro* Zn(ii)-mediated hetero-complex binds Zn(ii) at sub-nanomolar concentrations.^10^ To fully understand zinc speciation in such a system it is essential that the individual stability constants, *i.e.*, the Zn(ii) affinities of each protein and the affinity of the apo-protein for the Zn(ii)-loaded partner, are known.

The Zn(ii) affinities (*K*_Zn_ values) of Atx1 and PacS_N_ have previously been measured using a competition assay with the chromophoric chelator RhodZin-3 (Table 1).^3*a*^ When the apo-protein is present in excess of Zn(ii), both Atx1 and PacS_N_ form Zn(ii)-bridged homo-dimers, with the affinity of the apo-protein for the Zn(ii)-loaded protein (*K*_Zn2_) in the low μM range (Table 1).^3*a*^ Homo-dimerization is not surprising given that Zn(ii) favours tetra-thiolate sites.^11^ To obtain structural insight into Zn(ii)-induced dimerization we crystallized the cyanobacterial Atx1 in the presence of half an equivalent of Zn(ii). The structure of the Zn(ii)-bridged dimer (Fig. 1) is similar to that of a side-to-side Cu(i)_2_–Atx1 dimer^1*b*^ (rmsds for C^α^ atoms of ∼0.6 Å for the two monomers), and also of HAH1 dimers linked by single Cu(i), Hg(ii) and Cd(ii) ions.^1*a*^ The tetra-nuclear Cu(i) cluster of the Cu(i)_2_–Atx1 dimer is replaced by a single Zn(ii) ion coordinated by Cys12 and Cys15 from each monomer in a tetrahedral arrangement, with Zn(ii)–S^γ^ distances of ∼2.3 Å and S^γ^–Zn–S^γ^ angles of 102–122°. The interaction interface remote from the metal site of Atx1 is largely conserved in complexes bridged by both Cu(i) and Zn(ii). Therefore, MBDs that form complexes with the metallochaperone in the presence of Cu(i) are expected to also form Zn(ii)-mediated complexes.

**Table 1:** Affinities for Zn(ii) (*K*_Zn_), of the apo-protein for the Zn(ii)-protein (*K*_Zn2_), of the Zn(ii)-protein for the apo-partner (*K*hetZn), and also of the Cu(i)-protein for the apo-partner (*K*hetCu)^*a*^

Protein	*K*_Zn_ (M^–1^)	*K*_Zn2_ (M^–1^)	*K* het Zn (M^–1^)	*K* het Cu (M^–1^)
Atx1	(6.9 ± 0.6) × 10^8 ^ ^*b*^	(1.5 ± 0.2) × 10^5 ^ ^*b*^	(4.0 ± 2.0) × 10^6^	
PacS_N_	(4.2 ± 0.4) × 10^7 ^ ^*b*^	(2.6 ± 0.3) × 10^5 ^ ^*b*^		∼10^4 ^ ^*c*^
HAH1	(2.0 ± 0.1) × 10^8^	(2.0 ± 0.6) × 10^4^		
MNK1	(5.9 ± 0.2) × 10^8^		(2.0 ± 0.8) × 10^7^	∼10^5 ^ ^*d*^

^*a*^Zn(ii) affinities were determined in 25 mM 4-(2-hydroxyethyl)piperazine-1-ethanesulfonic acid pH 7.4 plus 100 mM NaCl.

^*b*^From ref. 3*a*.

^*c*^From ref. 2*b*.

^*d*^From ref. 2*d*.

**Fig. 1:**
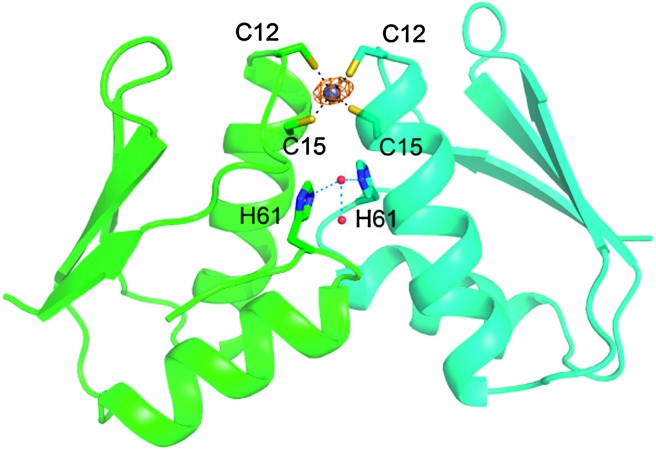
The Zn(ii)–(Atx1)_2_ crystal structure highlighting metal–ligand bonds and the hydrogen bonding network at the dimer interface involving His61, two water molecules and Ala59 (backbone carbonyl, not shown). The zinc ion and the oxygen atoms are shown as grey and red spheres respectively. The anomalous density for zinc is shown (orange mesh) contoured at 5*σ*.

To investigate Zn(ii)-mediated hetero-complex formation the removal of Zn(ii) from Zn–RhodZin-3 by PacS_N_ in the presence of a fixed amount of Atx1 was studied (Fig. 2A and see ESI†). These data cannot be explained by the presence of Zn(ii)-loaded protein alone (upper line in Fig. 2A), and fitting to eqn I (see ESI†) allows an affinity of apo-PacS_N_ for Zn(ii)–Atx1 (*K*hetZn) of (4.0 ± 2.0) × 10^6^ M^–1^ to be determined. The overall stability of the Atx1–Zn(ii)–PacS_N_ hetero-dimer (*K*_Zn_ *K*hetZn) is higher than that of the Zn(ii)–Atx1 homo-dimer (*K*_Zn_ *K*_Zn2_) (Table 1), presumably due to additional electrostatic interactions remote from the metal site, as proposed by NMR studies of the Atx1–Cu(i)–PacS_N_ hetero-complex.^2*b*^ This high affinity will enable significant amounts of the Atx1–Zn(ii)–PacS_N_ complex to form at Zn(ii) concentrations at which the MBD of the zinc exporter (ZiaA_N_) is 50% Zn(ii)-loaded (∼10^–10^ M), and at low μM concentrations of apo-Atx1 (Fig. 2C). Moreover, the Zn(ii)-mediated complex is 2–3 orders of magnitude tighter than that bridged by Cu(i),^2*b*^ due to the preference of Zn(ii) over Cu(i) for tetra-thiolate coordination.

**Fig. 2:**
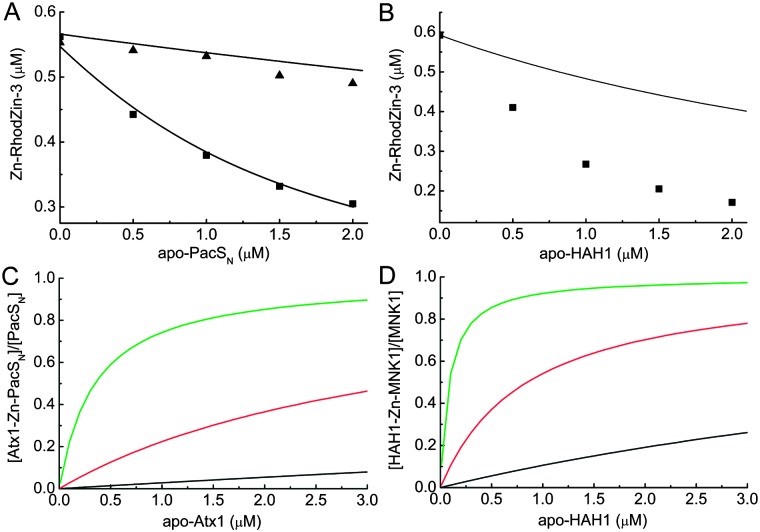
Titrations of apo-PacS_N_ (A, ■) and apo-HAH1 (B) into a mixture containing 1 μM Zn(ii), 10 μM RhodZin-3 and 1 μM of either Atx1 (A) or MNK1 (B). Also shown in (A) is the titration (▲) of apo-PacS_N_ into 0.55 μM Zn–RhodZin-3 in the presence of an excess (9.5 μM) RhodZin-3. The lower line in A represents the fit of the data to eqn I (see ESI†), yielding a *K*hetZn of (2.9 ± 0.4) × 10^6^ M^–1^. The upper line in A and the line in B represent the calculated values for the corresponding titration if hetero-complex formation does not occur. The agreement in (A) with the actual data in the absence of Atx1 (▲) is excellent. Also shown is the fraction of the MBD [PacS_N_ (C) or MNK1 (D)] that will form a Zn(ii)-bridged complex with the corresponding metallochaperone [Atx1 (C) or HAH1(D)] as a function of the concentration of apo-Atx1 (C) or apo-HAH1 (D) at free zinc concentrations of 10^–11^ M (black line), 10^–10^ M (red line) and 10^–9^ M (green line).

In *Synechocystis*, a second P-type ATP-ase (CtaA) has been proposed to be involved in the copper trafficking pathway to the thylakoids. The phenotype of a CtaA mutant indicated a role in copper import,^4*a*^ but *in vitro* data support a similar function to that of PacS; copper export from the cytosol,^12^ although the two transporters are probably not redundant. The MBD of CtaA (CtaA_N_) interacts with Atx1 in a two-hybrid assay,^4*b*^ and CtaA_N_ has a Zn(ii) affinity ∼20 fold higher than that of PacS_N_.^3*a*^ Cu(i) transport by CtaA and PacS may be regulated at different zinc concentrations, possibly to achieve prioritization of copper to the two targets (cytochrome *c* oxidase and plastocyanin) under altered zinc nutritional states. Our suggestion that this involves Zn(ii)–Atx1 is consistent with the proposal that Atx1 binds Zn(ii) *in vivo*.^6^ A connection between zinc and copper metabolism has recently been suggested in the green alga *Chlamydomonas reinhardtii*, as copper responsive genes also respond to a shortage of zinc.^13^ The zinc-deficient cells accumulate copper by up to 50-fold but are functionally copper deficient (the copper is not accessible to plastocyanin), consistent with impaired copper transport.

The possibility of crosstalk between copper and zinc homeostasis *via* CXXC-containing domains in the *Synechocystis* proteins led us to explore Zn(ii)-mediated hetero-complex formation for a related Cu(i)-trafficking system from another organism. For this we chose HAH1 (human) and the first MBD of MNK (MNK1), whose interaction is well characterized.^2*d*^ The Zn(ii) affinities (*K*_Zn_ values) of HAH1 and MNK1, determined from competition assays with RhodZin-3,^3*a*^ are (2.0 ± 0.1) × 10^8^ and (5.9 ± 0.2) × 10^8^ M^–1^, respectively (Table 1). These values are up to one order of magnitude tighter than the weakest zinc site in metallothionein-2 (10^7.7^ M^–1^ at pH 7.4).^14^ The affinity of apo-HAH1 for the Zn(ii)-loaded protein (*K*_Zn2_) is in the high μM range (Table 1), while for MNK1 no Zn(ii)-bridged homo-dimer could be detected from competition with RhodZin-3. This is consistent with both HAH1 and MNK1 loaded with half an equivalent of Zn(ii) eluting as monomers from a gel-filtration column (Fig. S1, see ESI†), most likely as a mixture of apo and Zn(ii)-loaded forms.

The titration of apo-HAH1 into a mixture of Zn(ii), RhodZin-3 and MNK1 indicates the formation of a Zn(ii)-bridged hetero-complex (Fig. 2B), with an average *K*hetZn of (2.0 ± 0.8) × 10^7^ M^–1^ (eqn III, see ESI†). This is consistent with an estimated stability of this complex of greater than ∼10^7^ M^–1^ from gel-filtration experiments (see ESI†). The overall stability (*K*_Zn_ *K*hetZn) of the HAH1–Zn(ii)–MNK1 complex (1.2 × 10^16^ M^–2^) is ∼4-fold greater than that of Atx1–Zn(ii)–PacS_N_ (2.8 × 10^15^ M^–2^) and ∼3 fold tighter than that determined previously for the fluorescently-labeled HAH1–Zn(ii)–WLN4 complex (4.5 × 10^15^ M^–2^) using a FRET-based assay and an EGTA Zn(ii) buffering system.^10^

Our observation that the HAH1–MNK1 Zn(ii)-mediated interaction is ∼200-fold tighter than that mediated by Cu(i)^2*d*^ has particular importance given that MNK expression^8*b*^ and phosphorylation^8*a*^ increases in the presence of Zn(ii). The levels of free zinc vary in different human cell types, with an average steady-state concentration of ∼100 pM.^11^ The formation of such a tight Zn(ii)-complex indicates that at these zinc concentrations, and at apo-HAH1 concentrations of 1 μM, more than half of the MNK1 that does not coordinate Cu(i) may be bound to Zn(ii)–HAH1 (Fig. 2D). It thus appears that at low Cu(i) concentrations, the MBDs of the human copper-transporting ATP-ases can form Zn(ii)-mediated complexes with HAH1. MNK and WLN each have six MBDs, but not all form detectable complexes with HAH1 in the presence of Cu(i),^2c,e^ and presumably also not with Zn(ii). MNK1 and MNK4 favor complex formation with HAH1,^2c,15^ whilst MNK3 has much weaker affinities for HAH1 (ref. 15) and Cu(i).^7*b*^ It is not understood why six MBDs are required in humans, when only one or two are present in the corresponding bacterial and yeast transporters, but it has been suggested it may help stabilize interactions with HAH1.^16^ A separate (distinctive) role in sensing Zn(ii), and perhaps other metals, for some domains is an intriguing possibility. Transient changes in intracellular zinc concentration have a potential role in cell signaling and can be dissipated *via* so-called ‘muffling’ reactions.^17^ Increased Zn(ii) levels may affect the rate of Cu(i) transport by triggering conformational changes in the ATP-ase as a result of complex formation between the MBDs and the metallochaperone, and could provide a direct link between zinc-mediated signaling and copper transport.

Numerous potential connections between copper and zinc homeostasis have been identified as well as that already mentioned in *C. reinhardtii* (*vide supra*).^13^ For example, the gene for the Atx1 homologue (CopZ) is greatly downregulated under zinc limitation in *Pseudomonas protegens* Pf-5.^18*a*^ In *Enterococcus hirae*, CopZ replaces a single Zn(ii) ion in the CopY repressor with two Cu(i) ions.^18*b*^ The copper metallochaperone for the human Cu, Zn superoxide dismutase (CCS) binds Zn(ii), with CCS being a sensitive biomarker of zinc-induced copper deficiency.^19*a*^ Furthermore, zinc has been used to treat patients with Wilson's disease, a defect in WLN that results in copper overload.^19*b*^ In this study we provide evidence that CXXC-containing MBDs of Cu(i)-trafficking proteins can be directly involved in crosstalk between Cu(i) and Zn(ii) homeostasis in both prokaryotes and eukaryotes. Abnormal metal distributions, primarily in the brain, are associated with a range of neurodegenerative diseases and ageing.^20^ In particular, an imbalance between copper and zinc in humans has been linked to a series of neurological conditions resulting in aggression, post-partum depression and autism in children.^21^ This imbalance has been largely attributed to metallothionein dysfunction, but the direct effect of zinc concentration on the activity of copper transporters is another area that should be explored.

We thank staff at the Diamond Light Source for help with data collection. This work was supported by the Biotechnology and Biological Sciences Research Council (BBSRC) grant BB/E016529.

## References

[cit1] Boal K., Rosenzweig A. C. (2009). Chem. Rev..

[cit2] Banci L., Bertini I., Cantini F., Felli I. C., Gonnelli L., Hadjiliadis N., Pieratelli R., Rosato A., Voulgaris P. (2006). Nat. Chem. Biol..

[cit3] Badarau A., Dennison C. (2011). Proc. Natl. Acad. Sci. U. S. A..

[cit4] Tottey S., Rich P. R., Rondet S. A., Robinson N. J. (2001). J. Biol. Chem..

[cit5] Thelwell C., Robinson N. J., Turner-Cavet J. S. (1998). Proc. Natl. Acad. Sci. U. S. A..

[cit6] Dainty S. J., Patterson C. J., Waldron K. J., Robinson N. J. (2010). JBIC, J. Biol. Inorg. Chem..

[cit7] Yatsunyk L. A., Rosenzweig A. C. (2007). J. Biol. Chem..

[cit8] Hung Y. H., Layton M. J., Voskoboinik I., Mercer J. F. B., Camakaris J. (2007). Biochem. J..

[cit9] DiDonato M., Zhang J., Que L., Sarkar B. (2002). J. Biol. Chem..

[cit10] Van Dongen E. M. W. M., Dekkers L. M., Spijker K., Meijer E. W., Klomp L. W. J., Merkx M. (2006). J. Am. Chem. Soc..

[cit11] Maret W., Li Y. (2009). Chem. Rev..

[cit12] Raimunda D., González-Guerrero M., Leeber B. W., Argüello J. M. (2011). Biometals.

[cit13] Malasarn D., Kropat J., Hsieh S. I., Finazzi G., Casero D., Loo J. A., Pellegrini M., Wollman F. A., Merchant S. S. (2013). J. Biol. Chem..

[cit14] Krezel A., Maret W. (2007). J. Am. Chem. Soc..

[cit15] Arumugam K., Crouzy S. (2012). Biochemistry.

[cit16] Keller M., Benítez J. J., Klarin D., Zhong L., Goldfogel M., Yang F., Chen T. Y., Chen P. J. (2012). J. Am. Chem. Soc..

[cit17] Colvin R. A., Holmes W. R., Fontaine C. P., Maret W. (2010). Metallomics.

[cit18] Lim C. K., Hassan K. A., Penesyan A., Loper J. E., Paulsen I. T. (2013). Environ. Microbiol..

[cit19] Iskandar M., Swist E., Trick K. D., Wang B., L'Abbé M. R., Bertinato J. (2005). Nutr. J..

[cit20] Bolognin S., Messori L., Zatta P. (2009). NeuroMol. Med..

[cit21] Walsh W. J., Isaacson H. R., Rehman F., Hall A. (1997). Physiol. Behav..

