# Supplementary material for: Crosstalk between Cu(i) and Zn(ii) homeostasis via Atx1 and cognate domains
Source: Chem Commun (Camb). 2013 Aug 7;49(73):8000–2. doi: 10.1039/c3cc42709a (PMC3763678; doi:10.1039/c3cc42709a)
Supplement: Supplementary file 1 [file CC-049-c3cc42709a-s001.pdf]

Electronic Supplementary Material (ESI) for Chemical Communications
This journal is © The Royal Society of Chemistry 2013

SUPPLEMENTAL INFORMATION

Crosstalk between Cu(I) and Zn(II) homeostasis via Atx1-like domains

Adriana Badarau, Arnaud Baslé, Susan J. Firbank and Christopher Dennison*

Institute for Cell and Molecular Biosciences, Medical School, Newcastle University, Newcastle upon Tyne, NE2 4HH, U.K.

1. MATERIALS AND METHODS

Protein Purification, Reduction, Quantification and Analysis

The expression and purification of the metallochaperones and metal binding domains was performed as described previously.[1,2] In short, each protein underwent a two step purification: anion exchange and gel filtration. Before the final gel filtration step the proteins were treated with 50 mM ethylenediaminetetraacetic acid to ensure removal of any bound Zn(II). Purified proteins were reduced with 10 mM dithiotreitol, then transferred to an anaerobic chamber (Belle Technology, [O_2] << 2 ppm) and exchanged into 25 mM 4-(2-hydroxyethyl)piperazine-1-ethanesulfonic acid (Hepes) pH 7.4 plus 100 mM NaCl, and all subsequent experiments were performed under strict anaerobic conditions. The quantification of the fully reduced apo-proteins was based on free thiol concentrations determined with 5,5`-dithiobis-(2-nitrobenzoic acid), as described previously,[1,2] assuming two thiols for Atx1 and MNK1, and three thiols for $PacS_N$ and HAH1. The Zn(II) loaded proteins were prepared by incubating the fully-reduced apo-proteins with the corresponding amount of $ZnSO_4$ (from a 1 mM stock), and Zn(II) concentration in all solutions was checked using atomic absorption spectrometry as described previously.[3]

Determination of the Zn(II) Affinities and the Stability Constants of Zn(II)-Mediated Hetero-Complexes

Zn(II) affinities were measured using the competitive chelator RhodZin-3 in 25 Hepes pH 7.4 plus 100 mM NaCl.[3] Data were fit to a model considering a single species (ZnP, where P is the apo-protein) for MNK1 to obtain the Zn(II) affinity of the apo-protein (K_{Zn}) and two species (ZnP and ZnP_2) for HAH1 to determine both K_{Zn} and the affinity of the apo-protein for zinc-protein (K_{Zn2}). The affinity of apo-$PacS_N$ for Zn(II)-Atx1 was determined by titrating apo-$PacS_N$ into a mixture containing 1 µM Zn(II), 10 µM RhodZin-3 and 1 µM Atx1. The decrease in Zn-RhodZin-3 concentration was fit to equation (I), which assumes that the concentration of Zn(II)-$PacS_N$ formed under these conditions is much smaller than the total Zn(II) concentration;

$$[P] = \frac{([Zn]-A)(1+K_{Zn}^{het}A)}{K_{Zn}^{het}A} \qquad \text{(I)}$$

where

$$A = K_{Zn}\frac{[ZnL]}{K_{Rh}[L]-[ZnL]}\left([Atx1]-[Zn]+[ZnL]\right) \qquad \text{(II)}$$

and [P], [Atx1], [Zn] and [L] represent total $PacS_N$, Atx1, Zn(II) and RhodZin-3 concentrations, respectively, K_{Rh} and K_{Zn} are the Zn(II) affinities of RhodZin-3 and Atx1,[3] respectively, and K_{Zn}^{het} is the affinity of apo-$PacS_N$ for Zn(II)-Atx1.

A similar approach was used to determine the affinity of apo-HAH1 for Zn(II)-MNK1. However, as HAH1 and MNK1 have similar Zn(II) affinities (*vide infra*), significant amounts of Zn(II)-HAH1 are formed during the titration with apo-HAH1 and data cannot be fit to equation (I). In this case, for each titration point K_{Zn}^{het} values were calculated using equation (III) and averaged;

$$K_{Zn}^{het} = \frac{\left([MNK1]-M\left(1+K'_{Zn}\frac{[ZnL]}{K_{Rh}\left([L]-[ZnL]\right)}\right)\right)}{M\left(K'_{Zn}\frac{[ZnL]}{K_{Rh}\left([L]-[ZnL]\right)}\right)\left(M\left(K'_{Zn}\frac{[ZnL]}{K_{Rh}\left([L]-[ZnL]\right)}\right)+[P]+[ZnL]-[Zn]\right)} \qquad \text{(III)}$$

where

$$M = \frac{[MNK1]+\left(K_{Zn}\frac{[ZnL]}{K_{Rh}\left([L]-[ZnL]\right)}\right)\left([P]+[ZnL]-[Zn]\right)-[P]}{1+K_{Zn}K'_{Zn}\left(\frac{[ZnL]}{K_{Rh}\left([L]-[ZnL]\right)}\right)^2} \qquad \text{(IV)}$$

and [P] and [MNK1] represent total HAH1 and MNK1 concentrations, K_{Zn} and K'_{Zn} are the Zn(II) affinities of HAH1 and MNK1, respectively, K_{Zn}^{het} is the affinity of apo-HAH1 for Zn(II)-MNK1, and the remainder of the symbols are as defined earlier.

Protein Crystallization, X-Ray Data Collection, Structure Determination and Refinement

Atx1 (11 mg/mL) loaded with 0.5 equivalents of Zn(II) in 20 mM Hepes pH 7.0 plus 20 mM NaCl was crystallised from 30% (w/v) PEG 4000, 0.2 M $MgCl_2$ and 0.1 M tris(hydroxymethyl)aminomethane (Tris) pH 8.5 using the sitting drop method of vapor diffusion [250 nL protein mixed with 250 nL well solution]. Crystals were frozen using N-paratone oil as the

This journal is © The Royal Society of Chemistry 2013

cryoprotectant. Diffraction data were collected at 100 K on beamline I04 at the Diamond Light Source (Didcot, UK). X-ray fluorescence scans were performed to confirm the absence of copper and the presence of zinc in crystals. Data were processed and integrated using iMOSFLM and scaled with Scala.[4,5] The structure was solved by molecular replacement using Molrep[6] and 2XMT (Atx1) as the search model.[1] A final model was produced with iterative cycles of refinement (Refmac5) and model-building using COOT.[7,8]

Analytical gel filtration

Analytical gel filtration was performed using a Superdex 75 10/300 GL column (GE Healthcare), equilibrated in thoroughly deoxygenated 25 mM Tris pH 7.5 plus 200 mM NaCl at a flow-rate of 0.4 ml/min. The column was calibrated using Blue Dextran (2000 kDa), conalbumin (75 kDa), ovalbumin (43 kDa), carbonic anhydrase (29 kDa), ribonuclease A (13.7 kDa) and aprotinin (6.5 kDa) in the same buffer.

This journal is © The Royal Society of Chemistry 2013

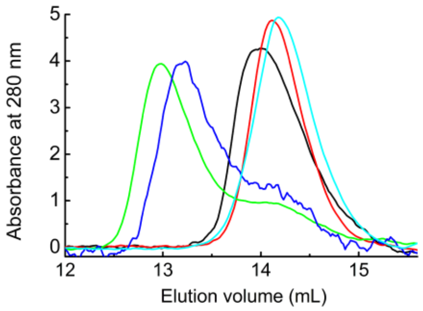

Figure S1. Plots of absorbance at 280 nm (arbitrary values) against elution volume from the gel filtration column in 25 mM Tris pH 7.5 plus 200 mM NaCl for MNK1 (20 µM, red line) and HAH1 (20 µM, black line) loaded with half an equivalent of Zn(II), apo-HAH1 (50 µM, cyan line), and an equimolar mixture of Zn(II), MNK1 and HAH1 (10 µM, green line; 2 µM, blue line). This mixture of Zn(II), HAH1 and MNK1 elutes at a molecular weight corresponding to a dimer even at 2 µM (blue line). Proteins are diluted ~ 5-fold during passage down the gel-filtration column and the stability of this complex can therefore be estimated to be greater than ~ 10^7 M^{-1}, in agreement with the competition experiments with RhodZin-3.

This journal is © The Royal Society of Chemistry 2013

Table S1: Data collection and processing statistics

Data collection [a]		*Refinement*	
Instrumentation	Diamond I04	Resolution range (Å)[b]	44.92 – 1.85 (1.95-1.85)
Wavelength (Å)	1.2822	TLS refinement	No
Space Group	P1	Anasitropic B-factors	No
Resolution range (Å)[b]	44.92 – 1.85 (1.95-1.85)	R_{work} (%)[d]	19.16
Unit cell parameters (Å, °)	*a* = 30.71	R_{free} (%)[c]	24.28
	b = 41.99	Rms Bond (Å)[d]	0.0129
	c = 45.24	Rms Angles (°)[d]	1.4878
	α = 83.34	No. of non-hydrogen atoms	1937
	β = 89.52	Average B factor (Å^2)	
	γ = 75.28		
Unique reflections	19967 (2475)	Protein	23.0
Multiplicity[b,c]	2.0 (2.0)	Water	30.5
[anomalous]	1.1 (1.1)		
Mean((I)/σ(I))[b,c]	5.1 (2.1)	Other solvent atoms	33.7
Completeness (%)[b,c]	91.5 (90.9)	Ramachandran outliers	0
[anomalous]	65.7 (63.4)		
R_{merge} (%)[b,c]	6.5 (19.5)	PDB accession codes	4A46

[a] Data is collected at the peak for the zinc edge. [b] Figures in parentheses are those for the highest resolution shell, as given. [c] Reflection statistics are as reported by SCALA.[4] R_{merge} is calculated as described in SCALA.[4] [d] Refinement statistics are as reported by REFMAC5.[7,8]

This journal is © The Royal Society of Chemistry 2013

REFERENCES

1. A. Badarau, S. J. Firbank, A. A. McCarthy, M. J. Banfield and C. Dennison, *Biochemistry*, 2010, **49**, 7798.
2. A. Badarau and C. Dennison, *J. Am. Chem. Soc.*, 2011, **133**, 2983.
3. A. Badarau and Dennison, *Proc. Natl. Acad. Sci. U.S.A.*, 2011, **108**, 13007.
4. P. Evans, *Acta Crystallogr. D Biol. Crystallogr.*, 2006, **62**, 72.
5. T. G. G. Battye, L. Kontogiannis, O. Johnson, H. R. Powell and A. G. W. Leslie, *Acta Crystallogr. D Biol. Crystallogr.*, 2011, **67**, 271.
6. A. Vagin and Teplyakov, *Acta Crystallogr. D Biol. Crystallogr.*, 2010, **66**, 22.
7. CCP4. *Acta Crystallogr. D Biol. Crystallogr.*, 1994, **50**, 760.
8. P. Emsley, B. Lohkamp, W. G. Scott and K. Cowtan, *Acta Crystallogr. D Biol. Crystallogr.*, 2010, **66**, 486.
